# Supplementary material for: Applications of the Microscale Thermophoresis Binding Assay in COVID-19 Research
Source: Viruses. 2023 Jun 25;15(7):1432. doi: 10.3390/v15071432 (PMC10386446; doi:10.3390/v15071432)
Supplement: Supplementary file 1 [file viruses-15-01432-s001.zip › viruses-2439560-supplementary.pdf]

viruses

MDPI

Supplementary Figure:

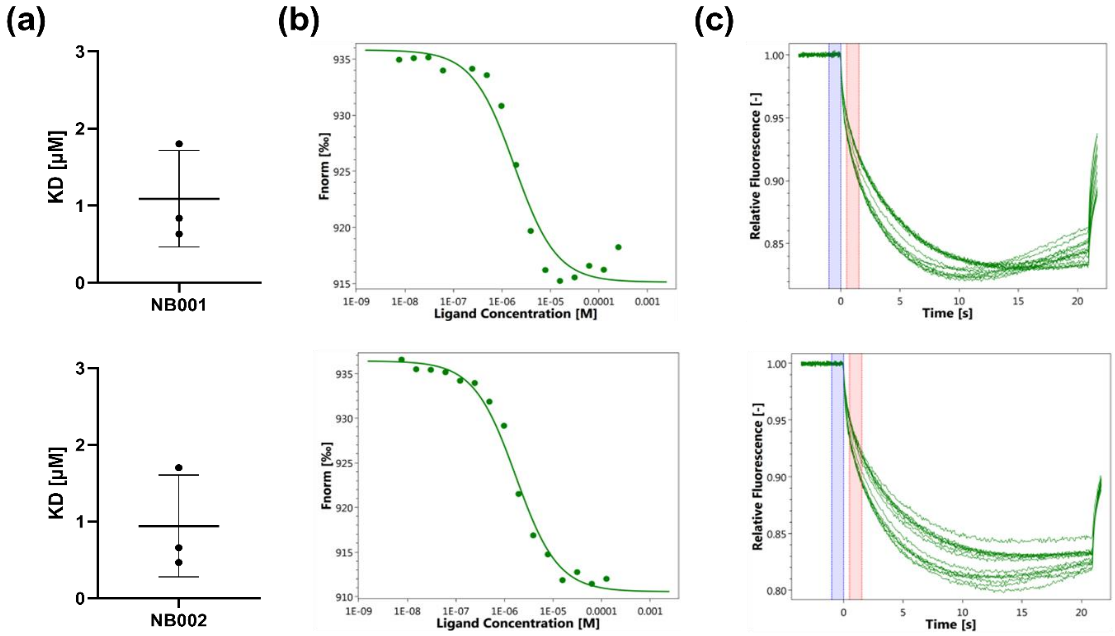

Figure S1. Binding of SARS-CoV-2-S1-RBD to the peptides NB001 and NB002. (**a**) The K_D for the binding of NB001 to RBD is 1.08µM, and that for NB002 is 0.94 µM. The following serial dilutions were used for the peptides: NB001R, 1mM-30nM; NB001 and NB002, 250µM-7.6nM. (**b**) Representative dose response curves (**c**) MST traces. The red bar indicates the selected time point for the dose response used to determine the K_D.
